# Temps d’attente prolongés aux services de consultation médicale: enjeux et perspectives pour des hôpitaux de Bukavu en République Démocratique du Congo

**DOI:** 10.11604/pamj.2018.29.173.13651

**Published:** 2018-03-26

**Authors:** Vicky Mulinganya, Florentin Asima, Patrick Mirindi, Hermès Karemere

**Affiliations:** 1 Ecole Régionale de Santé Publique, Université Catholique de Bukavu, République démocratique du Congo; 2 Département de Santé Publique, Université Officielle de Bukavu, République démocratique du Congo

**Keywords:** Temps d´attente, consultation médicale, hôpital, RD Congo, Waiting times, medical consultation, hospital, perspectives, Bukavu, South Kivu, Democratic Republic of Congo

## Abstract

**Introduction:**

Le temps d'attente en consultation devient un facteur d'insatisfaction des patients une fois qu'il est prolongé. L'objectif de l'étude est de mesurer les temps d'attente en consultation et d'en identifier les causes et conséquences dans une perspective d'amélioration de l'organisation des services.

**Méthodes:**

L'étude est descriptive transversale, réalisée à l'Hôpital Général Provincial de Référence de Bukavu et au centre hospitalier Biopharm. La revue documentaire, l'observation des temps d'attente et les entrevues individuelles avec des patients et des prestataires des soins ont été utilisées pour collecter les données. Le logiciel Epi Info 7.2.0.1. a servi à l'analyse des données quantitatives. Les données qualitatives ont été analysées par thème.

**Résultats:**

Plus de 70% des patients dans 3 grands services de l'HGPRB ont expérimenté un temps d'attente au-delà 30 minutes avant leur consultation. Pour plus de 40% des patients dans les 4 grands services du CH Biopharm, le temps d'attente a varié entre 0 et 15 minutes. Le service de gynéco-obstétrique de l'HGPRB a enregistré 56% des patients pour lesquels le temps d'attente est meilleur (0 à 15 minutes). Les deux principales causes des temps d'attente prolongés sont la lourdeur administrative des services et le retard des médecins aux consultations. Les conséquences redoutables sont la diminution de la fréquentation, la baisse des recettes et la fatigue des patients. La disponibilité du personnel qualifié, l'informatisation des services hospitaliers et la formation continue du personnel sont prioritaires pour réduire les temps d'attente.

**Conclusion:**

Les temps d'attente varient d'un hôpital à un autre et d'un service à un autre au sein d'un même hôpital en fonction de plusieurs facteurs. L'étude offre des pistes d'amélioration des temps d'attente en consultation médical.

## Abstract (en)

**Introduction:**

Prolonged waiting times for medical consultations become a factor in patient dissatisfaction. This study aimed to measure the waiting times for medical consultation and to identify the causes and the consequences from the perspective of improvement of the organisation of medical services.

**Methods:**

We conducted a cross-sectional descriptive study at the General Provincial Reference hospital in Bukavu and at the Biopharm Hospital Center. Data were collected using a literature review, the monitoring of the waiting times and individual interviews of patients and care providers. Quantitative data analysis was accomplished using Epi Info 7.2.0.1.software. Qualitative data were analyzed searching for themes.

**Results:**

More than 70% of patients in 3 major Departments at the General Provincial Reference Hospital in Bukavu waited more than 30 minutes before being seen. More than 40% of patients in the 4 major Departments at the Biopharm Hospital Center waited between 0 and 15 minutes. In the Department of Gynecology and Obstetrics at the General Provincial Reference hospital in Bukavu 56% of patients had shorter waiting times (0-15 minutes). The two main causes of prolonged wait times included administrative burden of medical services and physicians delay. Serious implications included a reduction in attendance, in medicinal recipes as well as patients’ fatigue. The availability of qualified health staff, the computerization of hospital services and continuous staff training are priorities to reduce waiting times.

**Conclusion:**

Waiting times vary from one hospital to another and from one department to another within the same hospital, depending on several factors. This study describes possible measures for improvement of waiting times for medical consultations.

## Introduction

Le temps d'attente est le délai avant qu'un patient reçoive un service de santé, soit une consultation, un diagnostic, un traitement ou une chirurgie [1]. Ce temps d'attente constitue un facteur d'insatisfaction des patients lorsqu'il est prolongé. Plusieurs études ont démontré que le temps d'attente des patients est un déterminant important dans la capacité d'une institution sanitaire à satisfaire la population qui utilise ses services [2, 3]. Bien encore, dans un environnement compétitif, le temps d'attente est perçu comme un critère décisif dans le choix des structures des soins à fréquenter [3]. Il est en effet difficile d'avoir une meilleure utilisation des services de santé si les utilisateurs ne sont pas satisfaits du temps qu'ils passent entre le moment où un patient entre dans la salle d'attente et le moment où il quitte effectivement l'hôpital [3]. Dans certains pays du monde, de longues files d'attente devant les portes de consultation médicale ont été observées [4-7], tant pour des services de médecine générale, des soins spécialisés, de réadaptation que de santé mentale [8]. Dans notre étude, nous considérons le temps d'attente sous plusieurs aspects selon l'itinéraire hospitalier du patient: durée de temps entre l'arrivée du patient à la salle d'attente et son entrée dans le bureau de consultation médicale; durée de temps entre la sortie du bureau de consultation et la réalisation d'un examen de laboratoire, d'une imagerie médicale ou d'un accès aux médicaments prescrits. Généralement, la mesure du temps d'attente aide à analyser le temps passé depuis l'admission dans la salle d'accueil jusqu'à la sortie de la consultation. Dans des études antérieures et de contextes différents, ce temps a été mesuré de plusieurs manières [5, 9]. Rendre ce temps d'attente satisfaisant constitue la préoccupation des gestionnaires dans plusieurs hôpitaux. A Bukavu, dans certains hôpitaux aussi bien publics que privés, l'obligation de passer beaucoup de temps, voire une journée entière, pour recevoir des soins en ambulatoire s'avère préjudiciable pour les patients. Certains d'entre eux retournent à leur domicile pour revenir le jour suivant ou un autre jour. Selon bon nombre de patients, adeptes de l'automédication ou de la prise de médicaments traditionnels (indigènes), aller rencontrer un médecin en consultation est une expérience pénible à cause de la longue file d'attente. C'est lorsqu'ils n'ont pas d'autre alternative face à leur problème de santé qu'ils se résignent à se rendre en consultation auprès des médecins, mettant ainsi à l'épreuve leur patience [10]. Cette situation semble constituer un sérieux problème d'organisation des services et provoque un accès réduit aux structures de soins de Santé avec un impact sur le financement des formations sanitaires, sur la santé de la population et sur la vie sociale des bénéficiaires des soins. En dépit de la pratique de certains médecins à la fois dans les hôpitaux privés et publics, les plaintes et l'insatisfaction émanant des usagers des services de consultations externes par rapport au long temps d'attente sont plus notées dans des hôpitaux publics que dans des formations sanitaires privées [11]. Le but du présent travail est de mesurer le temps d'attente au service de consultations externes dans un hôpital public comparé à un hôpital privé à Bukavu, d'en identifier les causes et d'en analyser les conséquences dans une perspective d'amélioration de l'organisation des services de consultation médicale afin de satisfaire les usagers.

## Méthodes

### Description du terrain d'étude

L'étude a ciblé deux hôpitaux de Bukavu; un hôpital public (l'hôpital général provincial de Bukavu) et un autre privé (le centre hospitalier BIOPHARM). Ces deux hôpitaux couvrent toute la population de Bukavu estimée **à 619.916 habitants en 2015** selon les statistiques de la mairie de Bukavu. Outre la perspective de comparer un hôpital public à un hôpital privé par rapport aux temps d'attente dans les services de consultation médicale, le choix de ces deux hôpitaux est justifié par leur réputation, leur fréquentation, leur accessibilité géographique et leur capacité d'accueil par rapport aux autres hôpitaux de la ville de Bukavu. En plus ces hôpitaux ont été choisis pour la facilité d'y accéder aux données hospitalières. Fondé en 1929, l'hôpital général provincial de référence (HGPR) de Bukavu a servi pendant de longues années comme hôpital de l'Etat avant d'être confié en 2013 à l'Archidiocèse de Bukavu qui en a fait, en plus, un hôpital d'enseignement pour les étudiants en médecine de l'Université Catholique de Bukavu (UCB). L'HGPR possède 16 pavillons et 22 chambres privées pour les hospitalisations dont deux servent aux consultations. Sa capacité théorique est de 500 lits mais seulement 350 lits sont montés. Ses principaux services sont: Gynéco-obstétrique, Pédiatrie, Chirurgie, Médecine interne, ophtalmologie, Dentisterie, Kinésithérapie, dermatologie, d'imagerie médicale et de consultations prénatales et préscolaires. Le Centre hospitalier Biopharm émane de la transformation d'un dépôt pharmaceutique italo-africaine (PIAF), œuvre de la coopération Congo-Italienne, crée en 1972. Il compte 72 lits et organise les services de chirurgie, de médecine interne, de pédiatrie, de gynécologie et obstétrique, de dentisterie, d'ophtalmologie, de kinésithérapie, d'imagerie médicale et de consultations prénatales et préscolaires. Des médecins généralistes et spécialistes y travaillent.

### Type d'étude

L'étude est de type descriptif transversal. Elle s'est déroulée à l'HGPR de Bukavu et au Centre hospitalier Biopharm au cours d'une période de 21 jours entre le 1^er^ Novembre et le 31 Décembre 2016. Elle décrit les temps d'attente en consultation médicale, indique des associations entre les variables liés au temps d'attente et génère des hypothèses explicatives de temps d'attente prolongées.

### Collecte des données

Pour collecter les données, nous avons fait recours à la fois à la revue documentaire, à l'observation participante et aux entrevues individuelles.

**La revue documentaire:** Elle a utilisé un outil de collecte d'information préalablement élaboré et comprenant les différents paramètres d'étude. Le fait pour le patient d'avoir été référé a constitué le critère d'inclusion [12] pour les consultations auprès des spécialistes. Les patients non référés ayant consulté chez le spécialiste étaient d'office exclus de l'étude. Les sources d'information ont combiné les registres des consultations des services de pédiatrie, médecine interne, chirurgie et gynéco-obstétrique, les carnets des patients reçus en consultation durant la période d'étude, quelques documents administratifs, le registre de consultation et le règlement d'ordre intérieur de chaque hôpital.

**Observation participante à l'aide d'un guide:** L'observation a consisté à observer d'une part le temps qui s'écoule entre l'arrivée du patient à la salle d'attente et d'autre part le temps que le patient passe dans le bureau de consultation médicale générale et spécialisée de manière à ne pas influencer le temps d'attente. Les autres temps au cours de l'itinéraire du patient en milieu hospitalier ont été observés et notés. Dans le cadre de cette étude, l'observation participante a aidé à comprendre de l'intérieur du système de santé les temps d'attente des usagers [13-15]. Des observateurs ont en effet été placés par salle d'attente des services de consultations, de laboratoire, de pharmacie et d'imagerie médicale et à l'aide d'un guide, ils ont noté dans un carnet leurs observations par rapport au temps que chaque malade ciblé par l'étude a passé à chaque étape. Ils ont également noté leurs observations par rapport à l'organisation des services, au respect des normes et règlements, à la manière dont le patient est accueilli en consultation, au comportement du personnel vis-à-vis des patients, à l'ambiance générale dans la salle d'attente, au niveau de satisfaction des patients, aux anomalies et dysfonctionnements des services et à toute autre élément pertinent. La stratégie de recherche a consisté à faire compléter les fiches de collecte d'information par des stagiaires par service et à assurer leur vérification par le chercheur principal. L'outil de collecte permettait de relever le temps mis par le patient à chaque étape de son itinéraire des soins dont par exemple le temps d'arrivé du patient à l'hôpital, le temps d'arrivé du patient au secrétariat du service sollicité, le temps d'arrivé du patient à la salle d'attente ou le temps d'arrivée du médecin pour la consultation. Ces informations ont été collectées par une infirmière et un stagiaire désignés par service. Au total, 768 patients ont constitué l'échantillon de la présente étude en raison de 384 patients par hôpital. Ils ont été sélectionnés par ordre de leur arrivée en consultation au cours de la période d'étude.

**Entrevues individuelles à l'aide d'un questionnaire semi-structuré:** Les entrevues individuelles ont été conduites auprès des médecins, des infirmiers et des patients ou de leurs proches pour déceler les causes et les conséquences des temps d'attente longs, mais aussi pour identifier des solutions spécifiques pouvant être mises en place afin de réduire ces temps d'attente prolongés. Un questionnaire a été élaboré à cet effet par catégorie des informateurs clés. Les services cibles de consultation ont concerné la Pédiatrie, la Médecine interne, la Gynéco-obstétrique et la Chirurgie. Les services d'imagerie médicale (échographie, radiographie, scanner), de laboratoire et de pharmacie ont également été explorés du fait qu'ils complètent la prise en charge des patients. Les langues utilisées lors des entrevues étaient le swahili, le mashi ou le français, selon la convenance du malade. Pendant la collecte, l'investigateur principal a assuré une supervision régulière par des visites sur terrain auprès des enquêteurs. La supervision avait consisté en la vérification de la façon dont les questionnaires étaient administrés et en la rectification des erreurs constatées.

**Analyse des données:** Des méthodes mixtes ont été utilisées pour analyser les données issues de la revue documentaire, de l'observation participante et des entrevues individuelles. Des données quantitatives ont été traitées à l'aide du logiciel EPI info 7.2.0.1 au travers des statistiques descriptives. Les informations qualitatives issues essentiellement des entrevues ont analysées par thème de manière à apporter des réponses aux questions spécifiques de l'étude. Le retour vers des informateurs clés a permis de confirmer ou d'infirmer les hypothèses générées lors de l'analyse des données. Les variables quantitatives analysées concernent essentiellement les caractéristiques des patients (notamment son âge), les caractéristiques des hôpitaux (dont le nombre du personnel) et les temps passés par le patient à différents étapes de son itinéraire au sein de l'hôpital. Les variables qualitatives analysées concernent également les caractéristiques du patient (dont le sexe, la profession, le niveau d'étude, etc), les caractéristiques des hôpitaux (dont le mode de paiement des soins, le motif de consultation.) et la perception des patients par rapport au temps d'attente prolongé (causes, conséquences, solutions).

**Considérations éthiques:** Le protocole de recherche avait reçu l'approbation de la commission universitaire de l'éthique de l'Université Catholique de Bukavu. Lors des entrevues, les principes du respect de la personne et de la confidentialité ont été garantis et observés, le consentement éclairé verbal pour chaque participant à l'étude a été obtenu systématiquement au travers un formulaire de consentement signé au préalable par chaque participant. La liberté d'adhérer à l'étude a été également respectée. Pour des patients analphabètes, un proche parent été sollicité pour réexpliquer le contenu du formulaire de consentement avant l'approbation d'apposer des empreintes digitales du participant à l'étude à l'endroit destiné à la signature du participant.

## Résultats

### Caractéristiques des hôpitaux observés

L'Observation a concerné les services de Pédiatrie, Médecine interne, Chirurgie et gynéco-obstétrique de chacun de deux structures hospitalières.

**Caractéristiques des hôpitaux pendant la période d'étude:** l'HPGRB compte 304 lits et 255 membres du personnel de plus que le CH BIOPHARM comme illustré dans le Tableau 1. Le nombre des médecins spécialistes est similaire (21 à l'HPGRB contre 20 au CH BIOPHARM) de même que le nombre de pharmaciens. Les infirmiers et les administratifs représentent dans les deux hôpitaux les catégories professionnelles ayant le plus grand nombre d'effectifs. Le nombre des patients reçus en consultation au cours de la période d'étude est plus important au CH Biopharm qu'à l'HGPRB (1728 contre 1034 patients) et la grande majorité n'est pas référée. On observe un nombre très faible (28) des patients référés au CH Biopharm contre un nombre plus important à l'HGPRB (507 patients). Aussi le nombre de consultations auprès des spécialistes est plus important au CH Biopharm (739) par rapport à celui noté à l'HGPRB (226).

**Tableau 1:** Comparaison nombre de lits, personnel et patients entre Novembre et Décembre 2016

Variables	HPGRB*	CH BIOPHARM**
**Nombre des lits**	376	72
**Personnel**	**n (% femmes)**	**n (% femmes)**
Médecins spécialistes	21(2)	20(3)
Médecins Généralistes	48(11)	9(1)
Infirmiers	148(70)	29(21)
Pharmacien	4(3)	4(3)
Laborantin	30(16)	5(1)
Technicien d'Imagerie	27(12)	1(1)
Administratif, Techniciens et Ouvriers	144(52)	41(15)
**Total Personnel**	**364(136)**	**109(45)**
**Patients**	**n**	**n**
Consultations durant la période d'étude	1034	1728
Consultations référées	507	28
Consultations non référées	527	1700
Consultations avec médecin généraliste	808	989
Consultations avec médecin spécialiste	226	739

* HGPRB: Hôpital Provincial Général de Référence de Bukavu

** CH: Centre Hospitalier

### Temps mesurés

Comparaison temps d'attente avant la consultation entre services des deux hôpitaux (Tableau 2)

**Tableau 2:** Proportions des patients par service, par hôpital et par temps d’attente avant la consultation

Temps d'attente (en minutes)	Chirurgie		Médecine interne		Gynéco-Obstétrique		Pédiatrie	
	HGPRB (n=91)	BIOPHARM(n=84)	HGPRB(n=94)	BIOPHARM(n=84)	HGPRB(n=81)	BIOPHARM(n=89)	HGPRB(n=89)	BIOPHARM(n=85)
0 à 5	5,5	3,6	3,1	8,4	30,8	11,2	9	18,8
6 à 10	3,3	28,6	2,2	15,5	8,7	18	2,2	21,2
11 à 15	4,4	10,7	3,1	17,8	16	14,6	4,5	18,8
16 à 20	0	7,1	4,2	8,4	9,9	9	0	9,4
21 à 25	2,2	10,7	2,2	15,4	4,9	7,9	2,2	5,9
26 à 30	3,3	1,2	2,2	10,7	2,5	4,5	7,9	9,4
> 30	81,3	38,1	83	23,8	27,2	34,8	74,2	16,5

**Figure 1:**
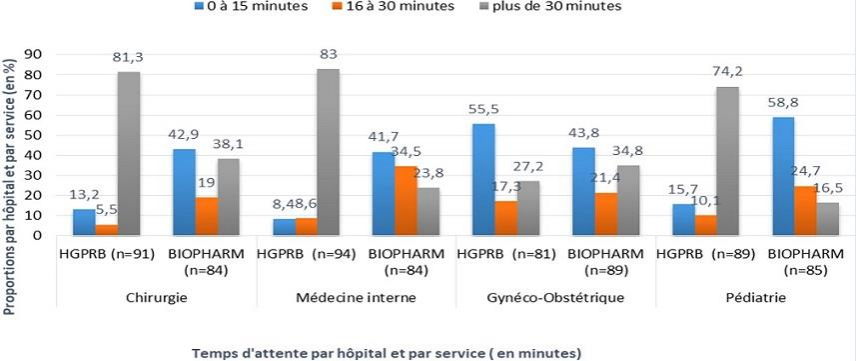
Proportions de patients par hôpital, par service et par temps d’attente en consultation

Les proportions importantes des patients ayant fait au plus 5 minutes avant la consultation sont observées dans les services de Gynéco-obstétrique de l'HGPRB (30,8%) et de Pédiatrie du CH Biopharm (18,8%). Au service de chirurgie du CH Biopharm 28,6% des patients attendent 6 à 10 minutes avant d'être vus en consultation médicale et pour le même temps, 21,2% des patients attendent en pédiatrie au CH Biopharm. Dans tous les services et dans les deux hôpitaux, plus de 20% de patients attendent plus de 30 minutes avant la consultation, excepté dans le service de Pédiatrie du CH Biopharm où seulement 16,5% des patients ont attendus plus de 30 minutes. Ces proportions des patients attendant plus de 30 minutes dépassent 70% en chirurgie HGPRB (81,3%), en médecine interne HGPRB (83%) et en Pédiatrie HGPRB (74,2%). Elles sont inférieures à 35% dans tous les services du CH Biopharm et dans le service de gynéco-obstétrique de l'HGPRB (Figure 1). Le temps d'attente dépassant 30 minutes a été observé pour une proportion dépassant 70% des patients dans 3 grands services de l'HGPRB dont la Chirurgie (81,3%), la Médecine interne (83%) et la pédiatrie (74,2%). Le temps d'attente variant entre 0 et 15 minutes a été le plus expérimenté par une proportion dépassant 40% des patients dans les 4 grands services du CH Biopharm dont la Chirurgie (42,9%), la médecine interne (41,7%), la gynéco-obstétrique (43,5%) et la pédiatrie (58,8%). Pour l'HGPR, seul le service de gynéco-obstétrique a enregistré plus de 40% soit 55,5% des patients ayant eu un temps d'attente meilleur (0 à 15 minutes) avant la consultation.

Comparaison des temps d'attente moyens par type de consultation ou de service paraclinique entre les deux hôpitaux (Tableau 3)

**Tableau 3:** Temps d’attente moyens mesurés pour des types de service dans les deux hôpitaux

Variable	HGPRB**		BIOPHARM	
	Nombre patients	Temps moyen (Minutes)	Nombre patients	Temps moyen (Minutes)
**Avant consultation**	385	30	388	30
Consultation médecin généraliste*	-	-	287	17
**Consultation en chirurgie**	95	20	95	12
**Consultation en Pédiatrie**	97	24	101	15
**Consultation en médecine interne**	99	15	97	13
**Consultation en Gynéco-obstétrique**	97	11	98	9
**Consultation médecin spécialiste**	385	13	101	16
**Laboratoire**	165	35	245	**80**
**Imagerie**	39	31	112	24
**Pharmacie**	125	11	341	5

* Données non disponibles

** HGPRB: Hôpital Provincial Général de Référence de Bukavu

**Figure 2:**
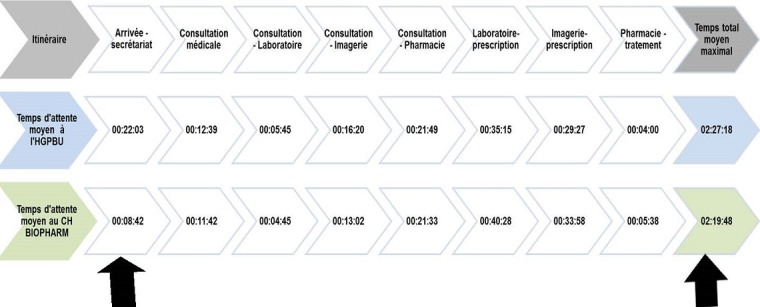
Temps moyens passé par le patient lors de son itinéraire hospitalier à l’HPGRB et au CH BIOPHARM

Le temps moyen d'attente avant la consultation est de 30 minutes. Les patients passent plus de temps à la consultation à l'HPGRB dans les services de chirurgie, de médecine interne et de pédiatrie par rapport Centre Hospitalier BIOPHARM. Partant du temps que le patient passe à l'hôpital, on constate que les patients passent plus de temps à l'HPGRB quand ils sont venus aux services de chirurgie, Médecine interne et pédiatrie que lors qu'ils sont venus au service de Gynéco Obstétriques. En parlant du Laboratoire, Imagerie et Pharmacie, nous remarquons que les patients trainent plus à l'HPGRB qu'au Centre Hospitalier BIOPHARM sauf pour le laboratoire où le temps d'attente au CH Biopharm est plus long (80 minutes). La Figure 2 renseigne que tout au long de l'itinéraire des patients dans les deux hôpitaux, le temps moyen semble similaire (02: 27: 18 à l'hôpital public-HPGRB contre 02: 19: 48 à l'hôpital privé-CH Biopharm) mais diffère de manière significative à l'accueil au secrétariat (00: 22: 03 à l'hôpital public-HPGRB contre 00: 08: 42 à l'hôpital privé-CH Biopharm).

### Causes des temps d'attente prolongés selon les personnes interrogées (Tableau 4)

La lourdeur des services (citées par 53,1% des personnes interrogées) et le retard des médecins (citées par 25,93% des personnes) constituent les principales causes des temps d'attente prolongées.

**Tableau 4:** Fréquence des causes des temps d’attente prolongés citées par les personnes interrogées

Causes du long temps d'attente	HPGRB*		BIOPHARM**	
	n	%	n	%
Mauvais accueil	1	0,4	13	8,7
Bruit	4	1,7	1	0,7
Cout élévé des soins	13	5,4	3	2
Couts élévé des médicaments	8	3,3	0	0
Lenteur au secrétariat des services	3	1,3	5	3,4
Lourdeur des services	127	53,1	94	63,1
Manque d'équipement	9	3,8	10	6,7
Manque d'orientation	2	0,8	1	0,7
Retard des médecins	62	25,9	3	2
Autres	10	4,2	19	12,8
**Total**	**239**	**100**	**149**	**100**

* HGPRB: Hôpital Provincial Général de Référence de Bukavu

### Conséquences des temps d'attente prolongés à l'HPGRB et au CH Biopharm (Tableau 5)

La diminution de la fréquentation de l'institution (citée par 40% des personnes interrogées à l'HPGRB et 36% Biopharm) ainsi que la fatigue des patients (citée par 16% des personnes interrogées à l'HPGRB et 8% Biopharm) sont les conséquences des temps d'attente prolongée dans ces structures.

**Tableau 5:** Principales conséquences des temps d’attente prolongées

Conséquences du long temps d'attente	HPGRB		BIOPHARM	
	n	%	n	%
Beaucoup de bruit et désordre dans la salle d'attente	3	12,0	4	16,0
Diminution de la fréquentation de l'institution	10	40,0	9	36,0
Baisse des recettes	3	12,0	6	24,0
Fatigue des patients	4	16,0	2	8,0
Frustration des patients	3	12,0	2	8,0
Mauvaise campagne sur l'institution	1	4,0	2	8,0
Fuite des cerveaux	1	4,0	0	0,0
**Total**	**25**	**100**	**25**	**100**

### Stratégies proposées pour réduire les temps d'attente prolongés (Tableau 6)

L'augmentation du personnel qualifié (52,7% des patients interrogés au CH BIOPHARM) et la rigueur à appliquer face au retard des médecins (26,7% des patients interrogés à l'HPGRB), sont les principales stratégies à mettre en place pour réduire le long temps d'attente.

**Tableau 6:** Fréquence des stratégies de réduction du long temps d’attente selon les patients

Stratégie	HPGRB		CH BIOPHARM	
	n	%	n	%
Augmentation du Personnel qualifié	62	21,7	108,0	52,7
Diminution du cout des soins	4	1,4	1,0	0,5
Disponibilité d'équipement	25	8,7	32,0	15,6
Rapprochement des services	4	1,4	0,0	0,0
Respect du circuit	1	0,4	0,0	0,0
Rigueur face au retard	103	36,0	4,0	2,0
Souplesse administrative	76	26,6	33,0	16,1
Autres	11	3,8	27,0	13,2
**Total**	**286**	**100**	**205**	**100**

## Discussion

L'étude avait comme objectifs de mesurer les temps d'attente en consultation médicale, d'identifier les causes et conséquences de long temps d'attente et de proposer des stratégies visant la réduction des temps d'attente en consultation médicale en comparant deux hôpitaux de la ville de Bukavu dont un public (l'Hôpital Général Provincial de Référence de Bukavu) et un autre privé (le Centre Hospitalier Biopharm). Nous discutons dans les paragraphes qui suivent les résultats obtenus en rapport avec les caractéristiques de deux hôpitaux, la mesure des temps d'attente, les causes et conséquences de longs temps d'attente, les stratégies proposées pour réduire les temps d'attente et les limites de l'étude.

### Caractéristiques de deux hôpitaux

Au cours de la période d'étude, l'HPGRB a enregistré moins de patients en consultation médicale que le Ch Biopharm (1034 contre 1728 patients) malgré le fait qu'il compte 376 lits (contre seulement 72 lits au CH Biopharm) et 255 membres du personnel de plus que le CH BIOPHARM. L'utilisation des services ne semble donc pas être lié au nombre du personnel travaillant dans un hôpital mais à d'autres facteurs parmi lesquels on peut évoquer l'accessibilité physique, géographique, culturelle et financière aux services; l'organisation des services ( horaires, gestion du flux des patients, temps d'attente); l'accueil des patients ( humain, physique et notamment les WC, douches,.); la qualité de la prise en charge ( disponibilité du personnel compétent, disponibilité des médicaments et possibilité de référence pertinente) [16-21]. Le nombre des médecins spécialistes est similaire (21 à l'HPGRB contre 20 au CH BIOPHARM) de même que le nombre de pharmaciens. Cela démontre naturellement que la qualité du personnel qualifié est meilleure au CH Biopharm, plus petit que l'HGPRB qui devait avoir un plus grand nombre de médecins spécialistes disponibles. Ce facteur pourrait contribuer à la meilleure utilisation des services de consultation médicale observée au CH Biopharm au cours de la période d'étude. On observe un nombre très faible (28) des patients référés au CH Biopharm contre un nombre plus important à l'HGPRB (507 patients). Les patients sont ainsi venus spontanément au CH Biopharm à la suite de la bonne perception des services de Biopharm qu'ils possèdent [22]. Aussi le nombre de consultations auprès des spécialistes est plus important au CH Biopharm (739) par rapport à celui noté à l'HGPRB (226).

### Mesure des temps d'attente

L'étude a recouru à une méthode de mesure des temps d'attente consistant à noter l'heure d'arrivée du patient, l'heure d'entrée et de sortie de la consultation médicale ou des autres services connexes. La méthode est appliquée dans un contexte où l'informatisation des systèmes de gestion des patients n'est pas encore implantée. Cette méthode manuelle est ainsi laborieuse, sujette à des erreurs (fatigue ou distraction de la personne affectée au comptage). Les temps d'attente très courts avant la consultation médicale (0 à 5 minutes) ont été observés dans les services de Gynéco-obstétrique de l'HGPRB (pour 30,8% des patients) et de Pédiatrie du CH Biopharm (pour 18,8% des patients). Il s'agit des records liés essentiellement aux aptitudes personnels des médecins qui dirigent ces services, selon la majorité des membres du personnel interrogé. Ces temps d'attente courts sont généralement observés lorsque les services sont mieux organisés (agenda bien tenu, liste des patients préalablement établie, patients contactés à l'avance) [1, 2,3] Les temps d'attente **dépassant 30 minutes** ont été observés pour une proportion dépassant 70% des patients dans 3 grands services de l'HGPRB dont la chirurgie (81,3%), la médecine interne (83%) et la pédiatrie (74,2%). La grande majorité des patients attendent plus longtemps (plus de 30 minutes) à l'HGPRB avant d'être reçus en consultation médicale tandis qu'une proportion dépassant 40% des patients dans les 4 grands services du CH Biopharm a mis moins de temps d'attente **(0 à 15 minutes)**. Dans plusieurs études, les temps d'attente ont dépassé 30 minutes [5, 23,24]. Le temps moyen idéal d'attente est de 22 minutes avant la consultation et n'est pas atteint dans les deux hôpitaux.

### Causes et conséquences des temps d'attente prolongées

### Causes

Les perceptions des patients et des prestataires des soins révèlent que les principales causes des temps d'attente prolongés dans les deux hôpitaux sont liées à l'organisation des services plutôt qu'aux caractéristiques des patients; elles sont donc institutionnelles. C'est en l'occurrence la lourdeur des services en termes de gestion du flux des patients (dans 53,1% des cas à l'HGPRB contre 63,1% des cas au CH Biopharm) et le retard des médecins (25,9% des cas à l'HGPRB contre seulement 2% au CH Biopharm). Les autres causes évoquées sont le mauvais accueil humain, le bruit, le coût élevé des soins, les coûts élevés des médicaments, la lenteur au secrétariat des services, le manque d'équipement et le manque d'orientation. La cause faisant la différence entre les deux hôpitaux est **le retard des médecins**, visiblement moins toléré par les patients qui sont nombreux à utiliser les services du CH Biopharm où les médecins sont plus ponctuels. Ces causes corroborent les résultats d'autres études [5, 23, 25-28].

### Conséquences

Les principales conséquences citées par ordre d'importance sont la baisse de la fréquentation de l'établissement hospitalier (40% des citations à l'HGPRB contre 36% au CH Biopharm), la baisse des recettes (16% à l'HGPRB contre 24% au CH Biopharm), le bruit et le désordre dans la salle d'attente (12% à l'HGPRB contre 16% au CH Biopharm) et la frustration des patients (12% à l'HGPRB contre 8% au CH Biopharm). Les autres conséquences sont la fatigue des patients, la mauvaise campagne sur l'institution avec risque de nuisance à la réputation de l'hôpital et la fuite des cerveaux, faute d'un paiement conséquent au personnel à la suite de la baisse des recettes. Il s'avère ainsi que de longs temps d'attente peuvent compromettre le développement d'un hôpital et hypothéquer son fonctionnement global [21, 29, 30]. Ce problème est donc à prendre en considération par les gestionnaires des hôpitaux afin d'améliorer la satisfaction des usagers et de faciliter un meilleur fonctionnement et une meilleure utilisation des services.

### Stratégies pour réduire les longs temps d'attente

Les résultats issus de la présente étude proposent les stratégies suivantes la formation continue du personnel (citées par 36% des prestataires à l'HGPRB), l'informatisation des services (20% à l'HGPRB et 32% au CH Biopharm), la motivation du personnel (24% à l'HGPRB et 36% au CH Biopharm), l'augmentation du personnel qualifié (16% à l'HGPRB et 20% au CH Biopharm), la rigueur dans la gestion des ressources humaines et notamment des médecins retardataires aux consultations externes (citée par 21,5% de l'ensemble des personnes interrogées), la souplesse administrative (22,2%) et la disponibilité des équipement (11,6%). Les autres stratégies concernent la baisse des coûts des soins, le rapprochement des services et le respect du circuit des patients. La mise en place de certaines stratégies peut nécessiter des moyens financiers qui limitent souvent l'organisation des hôpitaux et notamment ceux publics [31-34]. A défaut de réussir la mise en place de ces stratégies, le long temps d'attente est parfois exploité pour organiser des séances d'éducation sanitaire aux patients [24].

### Limites de l'étude

L'aperçue globale de la situation sur le temps d'attente des patients reçus aux services de consultation à Bukavu aurait nécessité une étude systématique de toutes les structures sanitaires remplissant des critères formels et sur tous les patients reçu en consultation. Les moyens n'auraient pas suffi pour le faire. Aussi, l'HGPRB est une structure médicale publique sous responsabilité et gestion de l'église catholique locale et se situe ainsi entre une structure publique stricte et une structure privée type, ce qui pourrait influencer les résultats de cette étude. Le système de tarification favorisant la prise en charge des pauvres avec un risque accru sur de longues files d'attente et des temps longs d'attente en consultation ambulatoire est appliqué à l'HGPRB. Ce risque semble minimisé dans l'étude d'autant plus que le CH Biopharm a enregistré plus de consultations que l'HGPRB durant la période d'étude en dépit des tarifs préférentiels de l'HGPRB. En ce qui concerne les patients sélectionnés, nous n'avons pas systématiquement interrogé tous les patients reçus aux services de consultation. Les services de consultation des structures fonctionnant 24heures sur 24 subdivisé en deux go, celui du jour allant de 7h30 à 17h00 et celui de garde allant de 17h00 à 8h00; nous avons focalisé nos enquêtes sur les patients admis entre 7h30 et 15h30 soit 8 heures au regard des contraintes (insécurité dans le milieu les soirées, difficultés de logement pour les enquêteurs et moyens financiers limités). Aussi nombreux médecins spécialistes de la structure privée ciblée n'arrivaient en consultation qu'en soirée faisant que certaines données sur le temps de prestations n'ont pas été habituellement prises, la majorité des participants à l'étude ont été reçu par les médecins généralistes.

## Conclusion

La présente étude a le mérite d'avoir démontré que les temps d'attente peuvent être mesurés dans un contexte non informatisée, que les temps d'attente varient d'un hôpital à un autre selon que l'hôpital est privé ou public; que ce temps peut également varier d'un service à un autre au sein d'un même hôpital en fonction de plusieurs facteurs dépendant à la fois du médecin (sa ponctualité) et de l'organisation de l'Établissement hospitalier (disponibilité des ressources humaines et matérielles). Les causes les plus fréquentes ainsi que les conséquences pertinentes des temps d'attente prolongés ont été identifiées pour chacun de ces deux hôpitaux et sont similaires. C'est essentiellement la lourdeur administrative et le retard des médecins en ce qui concerne les causes; l'insatisfaction des patients, la baisse de l'utilisation des services et la baisse de recettes en ce qui concerne les conséquences. Parmi les stratégies à mettre en place pour réduire les temps d'attente prolongée la priorité est accordée à la disponibilité du personnel qualifié, à l'informatisation des services hospitaliers et à la formation continue du personnel. Les principales recommandations aux équipes dirigeantes des deux hôpitaux sont ainsi la réorganisation des services, l'organisation de la formation continue du personnel, la sanction pour des retards aux heures de consultation, l'approvisionnement des services en outils informatiques, la standardisation des frais de consultation et l'installation des panneaux devant mieux orienter les patients.

### Etat des connaissances actuelle sur le sujet

- Les causes et conséquences des temps d'attente prolongés et les stratégies pour les réduire dans le contexte des pays développés.

### Contribution de notre étude à la connaissance

- La manière de mesurer manuellement le temps d'attente en milieu hospitalier dans un pays en voie de développement;
- La comparaison entre temps d'attente entre deux hôpitaux dont un public et un autre privé et la démonstration de la différence des temps d'attente liés à l'organisation des services.

### Conflits d’intérêts

Les auteurs ne déclarent aucun conflit d'intérêts.
